# Parasites Diversity, Abundance, Prevalence, and Richness Infecting *Didelphis aurita* (Didelphimorphia: Didelphidae) in the Atlantic Rainforest, Brazil

**DOI:** 10.3390/pathogens13090806

**Published:** 2024-09-18

**Authors:** Carolina Romeiro Fernandes Chagas, Cauê Monticelli, Caio Filipe da Motta Lima, Patrícia Locosque Ramos

**Affiliations:** 1 P. B. Šivickis Laboratory of Parasitology, Nature Research Centre, Akademijos 2, 08412 Vilnius, Lithuania; 2 Wildlife Coordination, Secretariat for the Environment, Infrastructure and Logistics (CFS/Semil), Av. Prof. Frederico Hermann Jr. 345, São Paulo 05454-010, Brazil; cmonticelli@sp.gov.br (C.M.); plramos@sp.gov.br (P.L.R.); 3 Post-Graduation Program in Wildlife Conservation, Federal University of São Carlos, Rodovia Lauri Simões de Barros, km 12, Buri, São Paulo 18290-000, Brazil; caio.lima@usp.br; 4 Departament of Veterinary Medicine, University of São Paulo, Av. Duque de Caxias Norte, 225, Pirassununga, São Paulo 13635-900, Brazil

**Keywords:** Brazil, *Cruzia*, endoparasites, opossum, *Sarcocystis*, São Paulo, South America

## Abstract

Parasites are key players in ecosystems, influencing population sizes and food webs, yet the impact of environmental factors on their diversity is not well understood. The Atlantic rainforest in Brazil, particularly the Parque Estadual das Fontes do Ipiranga (PEFI), exemplifies a biodiversity hotspot facing significant deforestation, housing diverse animal species such as the synanthropic Brazilian common opossum (*Didelphis aurita*), which serves as a reservoir for multiple zoonotic pathogens. In this study, we investigated parasite diversity, abundance, prevalence, and richness in free-living *D. aurita* in the PEFI, São Paulo, Brazil. From January 2015 to January 2017, 101 fecal samples of *D. aurita* were collected in two areas of PEFI, at the Instituto de Pesquisas Ambientais (IPA) and the Parque de Ciência e Tecnologia (Cientec), and analyzed using three different parasitological methods. In total, 99% of the samples were positive for at least one parasite. The most prevalent parasite belonged to the order Strongylida (82%), followed by *Cruzia* sp. (77%), the latter having a significantly higher prevalence at IPA. In contrast, Acanthocephala showed greater prevalence at Cientec. Co-infections were common, with some individuals harboring up to seven different parasites. Our findings reveal significant parasite diversity in the *D. aurita* population at PEFI, including both helminths and protozoan trophozoites, some of which are reported for the first time in this host species. Further research is essential for accurate species identification of the observed parasites.

## 1. Introduction

Parasites play a major role in the ecosystem, helping to regulate population size and food webs, controlling the survival rate in their host population, and contributing to the energy flow in different trophic levels [1,2]. Despite that, it is not completely understood how the environment can influence such diversity of parasites, an essential topic to be discussed in times of the One World One Health approach [3].

Some authors suggest the use of parasites as indicators of ecosystem health [4]. However, since environmental stressors and the responses to them can be considerably different depending on the hosts and the parasites, so using parasites as bioindicators should be done carefully [4]. Additionally, it is known that parasite diversity reflects host diversity [5], the size of forest fragments [6], host population size [7], rates of transmission, and host mortality [5]. Moreover, close contact with humans can also influence parasite diversity and their transmission in the environment [8].

Around the world, there are several places where biodiversity is threatened due to several human disturbances resulting in the extinction of species, habitat fragmentation, and population decline of animals. The ability to assist in protecting all these places is limited, which has led to the creation of biodiversity hotspots for conservation priorities [9], such as the two Brazilian biomes the Cerrado and the Atlantic rainforest [9,10].

The Atlantic rainforest extends into tropical and subtropical regions, in the Atlantic coast, from the south to the northeast of the country, resulting in highly heterogeneous environmental conditions, and consequently, a large biodiversity of species, that may include 1–8% of the world’s total fauna species [11]. However, it is located in the most populous region of Brazil, which has resulted in high deforestation due to the exploitation of wood, the establishment of cities, crop activities, hunting, among others [12]. Nowadays, the Atlantic rainforest has lost more than 85% of its native vegetation, which is composed mainly of small fragments with less than 50 ha [10,11].

One exception is the Parque Estadual das Fontes do Ipiranga (PEFI), the largest Atlantic rainforest remnant inside an urban area, in the São Paulo megalopolis. The PEFI covers around 495 ha. It was created in 1823, and in 1969 it became a Permanent Preservation Area, which indicates that this forest fragment should be permanently preserved due to its exceptional beauty, scientific and historic value, as well as sheltering fauna, and flora specimens that are considered threatened with extinction [13].

Recent studies performed in the PEFI confirmed a high diversity of animal species inhabiting the area, with more than 150 avian species [14], 25 native species of mammals [15,16], 22 species of anurans, and 24 species of reptiles [17]. One of the most common species in the PEFI probably is the Brazilian common opossum *Didelphis aurita* [15], a synanthropic species that can live in primary and secondary forests but is also found in places with high levels of deforestation and close to humans [18]. With a highly diverse diet, and synanthropic habits [19], *D. aurita* is constantly exposed to parasites and other pathogens, being considered reservoirs of several of them, including some with zoonotic potential, such as *Trypanosoma cruzi*, *Leishmania infantum*, *Toxoplasma gondii*, *Ancylostoma caninum*, and *Schistosoma mansoni* [20,21].

One of the most common parasites infecting *D. aurita* is *Cruzia tentaculata* [22,23]. However, several other parasites, mainly nematodes, have been reported in this host species, such as *Capillaria* sp., *Trichuris minuta*, *Heterostrongylus heterotrongylus*, *Metastrongylus* sp., *Travassostrongylus callis*, *Aspidodera* spp., *Subulura* sp., *Physaloptera* sp., *Turgida turgida*, *Acantocheilonema pricei*, *Echinocoleus auritae*, *Eucoleus fluminensis*, *Dracunculus fuelleborni*, *Gnathostoma turgidum*, *Gongylonema* sp., *Gongylonemoides marsupialis*, *Lagochilascaris turgida*, *Longistriata didelphis*, *Nematodirus didelphis*, *Strongyloides* sp., *Rhopalias* sp., *Oligacanthorhynchus microcephalus*, *Viannaia hamata*, *Sarcocystsis* sp., and *Eimeria* spp. [23,24,25,26,27,28,29,30,31]. Despite the high diversity of parasites infecting *D. aurita*, just a few studies have investigated their pathogenicity for this host species. For instance, it was shown that *Gnathostoma tugirdum* infections can result in severe stomach lesions; *Angiostrongylus cantonensis* can cause weakness, ataxia, and neurological abnormalities; *A. costaricensis* can result in peritonitis; and that *Heterostrongylus heterostrongylus* can be the cause of verminous pneumonia and mucous bronchiolitis [21,30,32].

In PEFI, there are different research, educational, and leisure institutions, such as the Instituto de Pesquisas Ambientais (IPA), Parque de Ciência e Tecnologia (Cientec), and the Coordenadoria de Fauna Silvestre (CFS). These institutions have brought attention to the PEFI and contributed to a large number of visitors every year. The presence of animals harboring pathogens with zoonotic potential that might represent a risk should be closely investigated. Given this, this study aimed to investigate parasite diversity, abundance, prevalence, and richness in free-living *D. aurita* in the PEFI.

## 2. Materials and Methods

### 2.1. Study Area

**Figure 1:**
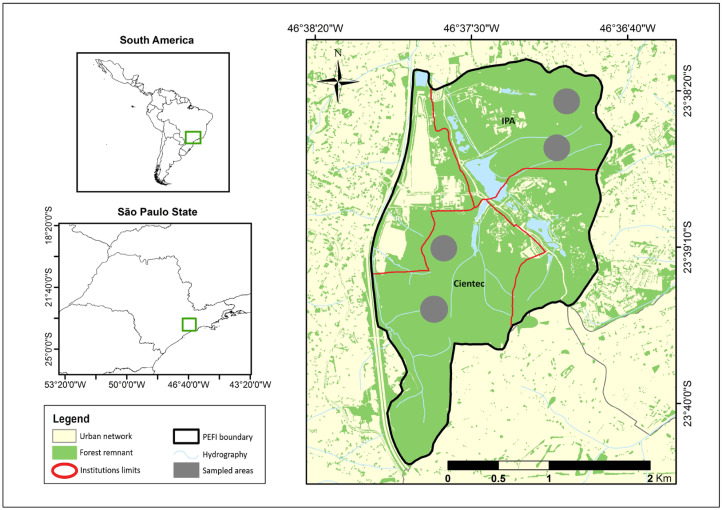
Study site showing the location of the Parque Estadual das Fontes do Ipiranga (PEFI) within South American and in the São Paulo state. Traps were set in the Instituto de Pesquisas Ambientais (IPA) and Parque de Ciência e Tecnologia (Cientec).

This study was conducted in two different areas of the PEFI, which have different levels of phyto-physiognomy. The first area was where the IPA is located. This place is characterized by areas with dense forests and homogeneous high-sized heterogeneous canopies (Figure 1). The second study area was the Cientec, which is characterized by a forest with sparse homogeneous canopies with discontinuous and sparse parts, as well as degraded areas (Figure 1). Both study sites are separated by a busy avenue. Detailed information about the study sites can be found in Monticelli et al. [15].

### 2.2. Animal Capture

*Didelphis aurita* individuals were captured in the study areas using two types of live capture traps: a box-trap (Sherman, size 30 × 7.5 × 9 cm) and a cage-trap (Tomahawk, size 45 × 20 × 20 cm). Both were baited with a mixture of sardines, cornmeal, bananas, peanut butter, and pineapple essence. The live traps were placed on the ground and at about two meters high, attached to understory branches. Pitfall traps were also arranged at the sampled points. Sampling took place from January 2015 to January 2017, for a total of 16 monthly research campaigns with a duration of five days each. Every area (IPA and Cientec) was sampled eight times, for four campaigns in the rainy season and four in the dry season. Captured animals were identified using ear tags with a predefined enumeration. Males and females were differentiated through the presence/absence of scrotum or marsupium, respectively. No sedation was used during capture and all animals were released a few minutes after being captured. All procedures of this investigation were approved by the pertaining environmental agencies under license SISBIO no. 45520 and SISGEN no. AE48610.

### 2.3. Sample Collection and Processing

Fecal samples were collected from the traps, stored in sterile cups with screw caps, and kept at room temperature until processing, which took between 1 to 4 h. All collected samples were processed using three different protocols as follows: (i) direct smear with saline solution, (ii) fecal flotation with sodium chloride (NaCl) solution with a specific gravity of 1.20 g/mL, and (iii) fecal sedimentation [33]. All preparations were microscopically analyzed at 100× magnification using an Olympus CX31 light microscope (Olympus, Tokyo, Japan). In the case of fecal sedimentation, a drop of 2% Lugol solution was used [33]. If a structure resembling a parasite was seen, it was analyzed at 400× magnification. The entire preparation was microscopically checked. Parasite stages were identified to the best of our ability, using the available literature [22,33,34,35,36].

If a sample was identified to be positive for coccidian oocysts (i.e., *Eimeria* spp. and *Cystoisospora* spp.), the sample was incubated for the sporulation of the oocyst and identification of parasite genera [33,35]. Briefly, this was performed by adding 2.5% potassium dichromate solution to the sample until it was fully immersed, gently mixed, and kept at room temperature in the dark for three to five days. After that, the samples were processed using the fecal flotation method mentioned above. The number of sporocysts in the oocysts and the number of sporozoites in each sporocyst were counted and used to identify the parasite genus [33,35].

### 2.4. Statistical Analysis

We calculated the abundance, prevalence, and richness of parasites in *D. aurita*. Abundance was considered as the number of hosts infected by each parasite with one or more individuals of a particular parasite taxon in each fecal sample. Prevalence was considered as the proportion of hosts infected with one or more individuals of a particular parasite taxon in each fecal sample relative to the total number of hosts sampled [8]. To compare the prevalence between the study sites we used the Chi-square test and considered an alpha value of 0.05. Richness was considered as the number of different parasite types per sample [8]. Therefore, we calculated one richness value for each individual host and subsequently presented the descriptive statistics with the distribution of richness values in the sampled population. All statistical analyses were conducted using R software [37].

## 3. Results

Samples from 101 individuals were evaluated during the study. In total, 52 were from the IPA area and 49 were from the Cientec area. Of those 67 were females and 34 were males. Except for one, all other analyzed samples (*n* = 100, 99%) were positive for at least one parasite, the only negative sample was collected at the IPA. All samples collected in the Cientec were positive. Eggs of helminths were detected in all positive samples, and protozoan parasites were present in 58.4% (59/101) of the samples (Table 1).

**Figure 2:**
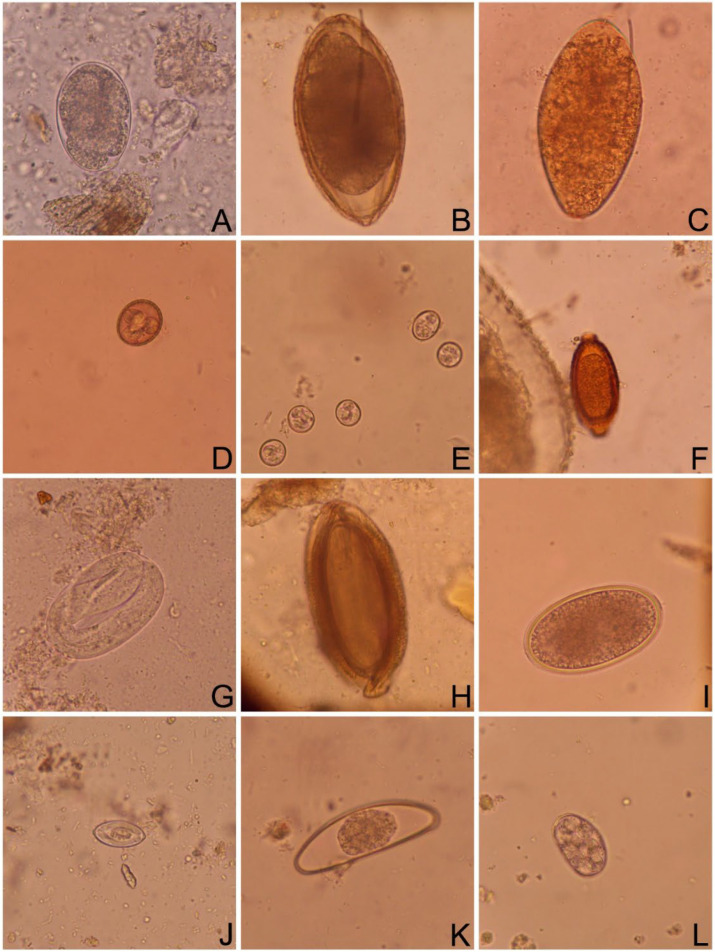
Helminth eggs (**A**–**C**,**F**–**I**,**K**) and coccidian oocysts (**D**,**E**,**L**) and sporocyst (**J**) found in *Didelphis aurita* feces sampled in the Parque Estadual das Fontes do Ipiranga (PEFI), São Paulo, Brazil. Strongylida (**A**), *Cruzia* sp. (**B**), Trematoda (**C**), *Eimeria* sp. (**D**,**E**), Trichuridae (**F**), Strongyloididae (**G**), Acanthocephala (**H**), Ascaridida (**I**), *Sarcocystis* sp. (**J**), Oxyuroidea (**K**), Adeleidae (**L**). Images were taken with the same magnification (400×), except for (**E**) (100×).

The most common helminth egg in the analyzed samples belonged to the order Strongylida (Figure 2A) and the second most common was *Cruzia* sp. (Figure 2B). In general, the prevalence of parasites found at both study sites was statistically similar. Nevertheless, certain variations were observed. Among the helminth infections, eggs of Acanthocephala (Figure 2H) were significantly more prevalent in the Cientec (*p* = 0.002), while the prevalence of *Cruzia* sp. was higher in the IPA (*p* = 0.003). The protozoan prevalence was slightly higher in the Cientec, which was the only study site where infections by *Sarcocystis* sp. (Figure 2J) were reported in this study. The prevalence of oocysts of *Eimeria* sp. (Figure 2D,E) was similar at both study sites (*p* = 0.365). A coccidian oocyst belonging to Adeleidae (Figure 2L), a pseudoparasite of *D. aurita*, was recovered from one of the analyzed samples in the Cientec (Table 1). No Cestode eggs were found during this study.

**Figure 3:**
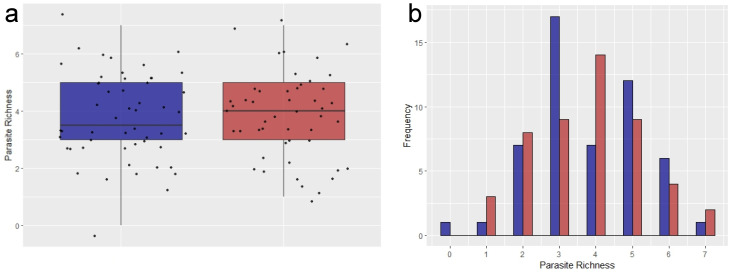
Parasite richness in fecal samples collected from *Didelphis aurita* in the Instituto de Pesquisas Ambientais (IPA)—in blue, and the Parque de Ciência e Tecnologia (Cientec)—in red, in Parque Estadual das Fontes do Ipiranga (PEFI), São Paulo, Brazil. Richness was considered as the number of different parasite types per sample. The parasite richness distributions of the two study sites are represented by a dot-boxplot (**a**) and a histogram (**b**).

Parasite richness was similar at both study sites, with the average number of parasites per individual being 3.79 and 3.78 for the IPA and Cientec, respectively; and the median values were 3.5 and 4.0 for the IPA and Cientec, respectively (Figure 3a). Except for one negative sample, all other tested samples were positive for at least one parasite. The highest number of parasites in co-infections was seven in the Cientec; while co-infections with two and four parasites were more commonly observed in the IPA, and with three and five parasites in the Cientec (Figure 3b).

## 4. Discussion

The main findings of this study were as follows: (i) a wide diversity and high prevalence of enteroparasites in *D. aurita* population from PEFI; (ii) a high number of co-infections in the studied population; (iii) Strongylida and *Cruzia* sp. were the most abundant helminths eggs found in the studied population, and *Eimeria* sp. was the most common protozoan; (iv) a significantly higher prevalence of Acanthocephala in the Cientec and of *Cruzia* sp. in the IPA. Unfortunately, it was not possible to perform a more detailed analysis of the parasitic forms (i.e., to collect measurements or perform molecular-based analysis) to identify the parasite species or even the parasite genus.

*Didelphis aurita* is known for harboring a wide diversity of parasitic infections, and several studies, in different parts of the Atlantic rainforest in Brazil, have pointed this out [21,22,23,24,34,38]. Even at different localities, the prevalence of parasitological infections is usually above 80%, and our study corroborates this with a prevalence of 99% [21,22,23,24,34,38]. We also reported a high number of samples positive for protozoan parasites (Table 1) when compared with the mentioned studies. One of the explanations for this might be the fact that we used three different parasitological methods to process the samples, while most studies are focused on helminths and collect adult worms for species identification [23,39], or only one or two methods are used when fecal samples are processed which usually does not include the direct method [34,40,41].

Using different diagnostic methods is essential for the diagnosis of parasites, especially in wild animals [42]. Applying the direct method in the fecal sample processing routine allows the determination of protozoan infections since trophozoites are not always clearly seen when other methodologies are used, but they can be easily recognized by their movements in the direct smears [33], even at lower magnification (100×). In this study, trophozoites of flagellated protozoa were present in 17 individuals (Table 1). These parasites are usually fragile, dying when other diagnostic methods are used, highlighting the importance of using techniques that could specifically target them. It is worth mentioning that the high number of co-infections detected, even though commonly reported in *D. aurita* [22,23], might also be because we applied three different diagnostic methods.

The most common helminth egg found in this study belonged to the Strongylida order (Table 1, Figure 2A). Using molecular methods, *Ancylostoma* sp. was previously reported in *D. aurita* individuals from another state in Brazil, and also in high prevalence (83.7%) [22]. Another study, using microscopical analysis of fecal samples, also reported the presence of Ancylostomatidae eggs, however, in a low prevalence with only two individuals being positive [24]. Eggs belonging to the families and different genera of the Strongylida order are morphologically similar and might be undistinguishable using only morphological analysis. Parasites of the Strongylida order seems to be the most commonly parasites found in *Didelphis* spp. with several studies reporting infections by *Heterostrongylus*, *Metastrongylus*, *Mammomonogamus*, *Longistriata*, *Nematodirus*, *Travassostgrongylus*, and *Viannaia* [26,27,34,43]. Unfortunately, we could not identify this parasite until lower taxonomic levels, which usually requires a combination of an integrative approach including morphological analysis of adult worms and molecular techniques.

We reported a high prevalence of *Cruzia* sp. (Table 1, Figure 2B) and most likely we were dealing with *Cruzia tentaculata* infections, which is one of the most common helminths parasitizing *D. aurita* that have been reported in the Southeast region of Brazil, where this study was conducted [22,23,24,34]. This parasite has been frequently reported in *Didelphis marsupialis*, *Didelphis virginiana*, and *Philander opossum* [41,44,45] as well. In our study, *Cruzia* sp. was the second most prevalent parasite, being more frequently found in the IPA (*p* = 0.003) (Table 1). This parasite has a direct life cycle and, most likely, eggs which are eliminated in more humid places and during the wet season are more likely to survive in the environment and reach the next host [36,41]. The IPA is an area with a dense forest and is more likely to have higher humidity than the Cientec, which has a sparse and discontinuous forest, providing more exposure to sun and other environmental conditions resulting in less humid areas for the eggs to remain viable in the soil for longer periods.

Trematode eggs were the third most found at both study sites (Table 1, Figure 2C); however, their identification was not possible. A few genera of Trematoda parasites were reported in *Didelphis*, mainly with zoonotic potential, being *Duboisiella*, *Brachylaima*, and *Rhopalias* [21,23,24,46,47]. Most likely we are dealing with *Rhopalias* infections, since this parasite genus was already reported infecting *D. aurita* at the São Paulo state [24], additionally, the trematode eggs found in this study were morphologically distinct from those belonging to the *Duboisiella* and *Brachylaima* genus (see [34]). This highlights the importance of conducting other studies in the area focused on the identification of these parasites and confirmation of species involved in these infections. A few studies have reported the presence of trematode parasites; however, this might be mainly due to the methodologies used for sample processing. Trematode eggs are heavy, and sedimentation methods are the most indicated methodology for retrieving them from the fecal samples [33].

Eggs of Trichuridae were also relatively common at both study sites (Table 1). Most likely, we were dealing with parasites belonging to the subfamily Capillariinae due to the lemon shape of the eggs and not so-evident operculum at each pole (Figure 2F) [33,36]. Even though not as common as other nematodes, several species of Trichuridae have been reported in different species of *Didelphis* in the Americas, such as *Capillaria* sp., *Echinocoleus auritae*, *Eucoleus fluminensis*, *Trichuris minuta*, and *Trichuris didelphis* [24,26,27,43,44,46]. It is worth mentioning that future projects targeting the identification of adult Trichuridae worms in *D. aurita* should be conducted [26,39,40,41,42] since differentiation between species only by eggs morphology is difficult, if not impossible.

With a prevalence of 17%, Strongyloididae eggs were present at both study sites (Table 1, Figure 2G). These parasites are not commonly found in *Didelphis*, with *Strongyloides* spp. being previous reported in a similar prevalence (14%) of this study [22]. Due to the morphological features of the eggs found in this study (presence of larva, thin wall, and ellipsoid shape [22,36]) we believe to be dealing also with *Strongyloides* spp. infections.

Acanthocephala parasites were also among the helminths found in our study, with a significantly higher prevalence in the Cientec (Table 1, Figure 2H). Even though several species of Acanthocephala have been described in *Didelphis*, they were later synonymized [48,49]. The Acanthocephala parasite found in our study most likely is *Oligacanthorhynchus microcephalus*. This parasite was originally described in Brazil, in the philander opossum *Caluromys philander*. Nowadays, its wide geographical distribution is already recognized, and infections in different locations in Brazil, Mexico, and the United States of America have been reported [46,48,49,50]. *Oligacanthorhynchus microcephalus* has an indirect life cycle, with the millipede *Narceus americanus* being the only intermediate host implicated in its life cycle until now [48]. However, this species of millipede does not occur in Brazil, so it is reasonable to believe that the intermediate host of *O. microcephalus* is still unknown in the country [48,49]. The vegetation differences at both study sites might indicate that some arthropod groups used as intermediate hosts of *O. microcephalus* are more commonly found in the Cientec. A detailed study targeting the microfauna in the area should be conducted to elucidate the life cycle of this parasite and which factors might be influencing this high prevalence.

Several species of helminths belonging to the Ascaridida order have been reported in opossums of the genus *Didelphis*, such as *Aspidodera*, *Cruzia*, *Subulura*, and *Lagochilascaris* [24,26,27,43,51]. The analysis we performed was based only on morphological features of eggs found in the feces of *D. aurita*. From this point of view, the Ascarididae eggs found (Figure 2I) do not resemble the eggs of any of the mentioned genera. Due to that, we prefer not to link this egg to any genus and use the order name to refer to them [36].

In the studied population of *D. aurita*, only two individuals were found to have eggs of Oxyuroidea parasites (Figure 2K), making it one of the least common helminth eggs. These eggs are elongated, have a thick shell, and are flattened on one side [36]. From our knowledge, this might be the first report of Oxyuroidea nematodes in *D. aurita*, which is worth a deeper investigation. These finding might lead to the question if this is not the result of contamination, since these parasites also infect rodents and other marsupials [25], which are common in the study areas [15]. However, as part of another study, we also processed several samples from small rodents and marsupials (*n* = 134), and eggs of Oxyuroidea were found in only two of them (1.5%) (C.R.F. Chagas person. comm.).

Regarding enteric protozoan parasites, *Eimeria* sp. is the most common protozoan found in *D. aurita* and other members of Didelphidae [22,24,44,52]. In this study, this was the protozoan with the highest prevalence (Table 1, Figure 2D,E). However, because not all studies applied methodologies for specifically retrieving these parasites, as well as for the identification of coccidian oocysts (i.e., sporulation using 2.5% potassium dichromate solution), these parasites can go unnoticed. There are several species of *Eimeria* described in *D. aurita*, identification of the *Eimeria* parasite found in this study would require a detailed morphological analysis, which was not conducted in the present study [53].

Some protozoan parasites receive a lot of attention for being highly pathogenic for humans and domesticated animals, as well as zoonotic potential, which is the case of *Trypanosoma*, *Plasmodium*, *Leishmania*, *Giardia*, *Toxoplasma*, among others [54,55]. However, other protozoan parasites are poorly investigated, resulting in poor knowledge of their prevalence and pathogenicity to their hosts, a scenario commonly seen in intestinal protozoan parasites of wild animals. In this study, we found a few individuals from both study sites with trophozoites of flagellated (Mastigophora) and ameboid (Sarcodina) protozoan parasites (Table 1). These parasites can be easily seen when the direct method is used for the parasitological diagnosis of parasites, mainly due to their characteristic movements. However, they can easily go unnoticed when other methods are applied, resulting in an underestimation of their prevalence. From our knowledge, *Tetratrichomonas didelphidis* is the only intestinal flagellated protozoan reported in *D. albiventris* and *D. marsupialis* in Brazil [56,57]. Unfortunately, other techniques, such as permanent staining, morphometry, electron microscopy, and molecular biology, are necessary to confirm parasite species. Yet, this might also be the first reported of ameboid (Sarcodina) protozoan parasites in *Didelphis*.

*Didelphis aurita* is considered one of the most well-adapted animals to the urban environments within Didelphidae occurring in Brazil. It can also have a high prevalence of zoonotic infectious agents, including parasites [20]. This highlights the importance of investigating such agents in different remnant fragments of the Atlantic rainforest, especially those close to humans, which is the case of PEFI. In this study, we could confirm the presence of *Sarcocystis* sp. in two individuals sampled in the Cientec (Table 1, Figure 2J). This is a known zoonotic agent that can cause severe disease in humans, especially in immunocompromised patients [20]. More studies should be conducted to better understand the distribution of these parasites in *D. aurita* and if this might represent a risk for the population visiting the area. In general, oocysts tend to be resistant and survive in the environment for long periods of time. In the case of *Sarcocystis* sp., oocyst walls are fragile, disintegrating during the passage through the intestine, and free sporocysts are released into the environment (which was the form found in this study, see Figure 2J). As long as they are in favorable conditions (cool to freezing temperatures), these sporocysts can remain viable for months but desiccation might kill them [58]. A drier environment is expected to be found in the Cientec, in contrast with the higher humidity encountered in the IPA. In that sense, it would be expected that a higher prevalence of *Sarcocystis* sp. would be found in the IPA, but no infections by this parasite were detected in this area. The reason why we only found *Sarcocystis* sp. in the Cientec is unclear.

One of the analyzed samples had oocysts of Adeleidae protozoan (Figure 2L), which, after sporulation, are characterized by the presence of several roundish sporocysts [59]. These protozoan parasites infect invertebrates, and they are commonly found in fecal samples of host predators, including *D. aurita* [59]. With an omnivorous diet that includes invertebrates, finding oocysts of Adeleidae is not a surprise.

The use of an integrative approach involving classical parasitology, with morphological and morphometric analysis and identification of adult worms combined with molecular techniques is essential in parasitological studies, especially when wild animals are involved [60]. Unfortunately, in this study, we were not able to apply all these techniques, resulting in some limitations, such as the impossibility to determine parasite species. Additionally, helminthological studies require the analysis of adult helminths, and to collect them it is necessary to euthanize several individuals, which was not performed.

## 5. Conclusions

This study provides valuable insights into the diversity, abundance, prevalence, and richness of parasites infecting the Brazilian common opossum, *Didelphis aurita*, in PEFI. The high diversity and prevalence of enteroparasites, including a significant number of co-infections, underscores the importance of this species as a reservoir for various parasitic infections, some of which might hold zoonotic potential, which should be carefully investigated in the future. The results demonstrate that environmental differences within PEFI can influence parasite prevalence, as seen with a higher prevalence of Acanthocephala in the Cientec area and *Cruzia* sp. in the IPA area. The use of three distinct diagnostic methods allowed for more comprehensive detection of protozoan parasites compared to previous studies, revealing a higher prevalence and diversity of these organisms. The findings further highlight the need for ongoing monitoring and detailed analysis, especially in areas close to human habitation, to better understand the ecological dynamics and mitigate potential public health risks. Future studies should aim to integrate molecular approaches with traditional parasitological methods for more detailed identification and understanding of parasite species and their life cycles.

## Figures and Tables

**Table 1:** Parasite diversity, abundance, and prevalence found in *Didelphis aurita* in the Instituto de Pesquisas Ambientais (IPA) and the Parque de Ciência e Tecnologia (Cientec), in the Parque Estadual das Fontes do Ipiranga (PEFI), São Paulo, Brazil. The number in parentheses represents the prevalence.

Parasite	Total (*n* = 101)	IPA (*n* = 52)	Cientec (*n* = 49)	*p*-Value
**Helminth (eggs)**				
Strongylida	83 (0.82)	40 (0.77)	43 (0.88)	0.245
*Cruzia* sp.	78 (0.77)	47 (0.90)	31 (0.63)	0.003 *
Trematoda	68 (0.67)	37 (0.71)	31 (0.63)	0.527
Trichuridae	44 (0.44)	23 (0.44)	21 (0.43)	1
Strongyloididae	17 (0.17)	10 (0.19)	7 (0.14)	0.691
Acanthocephala	13 (0.13)	1 (0.02)	12 (0.24)	0.002 *
Ascaridida	5 (0.05)	2 (0.04)	3 (0.06)	0.946
Oxyuroidea	2 (0.02)	1 (0.02)	1 (0.02)	1
**Protozoa (oocysts and trophozoites)**				
*Eimeria* sp.	49 (0.49)	28 (0.54)	21 (0.43)	0.365
Flagellated (Mastigophora)	17 (0.17)	7 (0.13)	10 (0.20)	0.505
Ameboid (Sarcodina)	4 (0.04)	1 (0.02)	3 (0.06)	0.568
*Sarcocystis* sp.	2 (0.02)	0 (0.00)	2 (0.04)	0.449
Adeleidae	1 (0.01)	0 (0.00)	1 (0.02)	0.976

(*) Parasites with significantly different prevalence values between the two study sites.

## Data Availability

Data are contained within the article.
